# Baltimore College of Dental Surgery

**Published:** 1852-07

**Authors:** 


*Baltimore College of Dental Surgery.*—It will be seen by the annual circular which accompanies this number of the Journal, that this Institution has been removed from its old quarters, in Lexington street, to a more central location, at the corner of Lombard and Hanover streets. The change of location is decidedly better for several reasons. In the first place, its position is more central; in the second, the *Infirmary* is in the college building, which, before, was not only detached, but at some distance from it; and in the last place, the accommodations furnished by the exchange, are greatly superior.

Those who have, from time to time, honored the institution with a visit, know that the old building was not wanting in the requisites necessary for *practical* and *theoretical* instruction, but an edifice better adapted to the purposes of a Dental College, than the present, could scarcely be planned. The building, erected at a cost of upwards of thirty thousand dollars, is entirely new. It has two lecture rooms which are unsurpassed for beauty, convenience and comfort, by any medical institution in the country. The *Infirmary* is well lighted, and has accommodation for half a dozen or more operating chairs. The *mechanical room,* forming the crowning beauty, if any distinction of adapted excellence can be drawn, extends the whole length of the building, eighty feet, is splendidly lighted from above; in addition to which, it is provided with gas fixtures, and abundantly supplied with hot and cold water. The *dissecting room* is admirably suited for the purposes for which it is designed.

In a word, the faculty have great reason to congratulate themselves on the exchange, as they are now prepared to offer greater facilities for instruction, at a much greater cost, it is true, to themselves, but without any additional expense to the student.

